# The Feasibility and Acceptability of the Adverse Childhood Experiences Questionnaire in Danish Antenatal Care—A Qualitative Study of Midwives’ Implementation Experiences

**DOI:** 10.3390/ijerph20105897

**Published:** 2023-05-20

**Authors:** Helle Johnsen, Vibeke de Lichtenberg, Eva Rydahl, Sara Mbaye Karentius, Signe Camilla Hjuler Dueholm, Majbritt Friis-Alstrup, Mette Grønbæk Backhausen, Katrine Røhder, Michaela Louise Schiøtz, Lotte Broberg, Mette Juhl

**Affiliations:** 1 Department of Midwifery and Therapeutic Sciences, University College Copenhagen, Sigurdsgade 26, 2200 Copenhagen N, Denmark; 2 Department of Gynecology and Obstetrics, Holbæk Hospital, Smedelundsgade 60, 4300 Holbæk, Denmark; scdu@regionsjaelland.dk; 3 Department of Obstetrics, Slagelse Hospital, Fælledvej 13, 4200 Slagelse, Denmark; mfu@regionsjaelland.dk; 4 Department of Gynecology and Obstetrics, Zealand University Hospital Roskilde, Sygehusvej 10, 4000 Roskilde, Denmark; mgb@regionsjaelland.dk; 5 Institute for Psychology, Copenhagen University, Øster Farimagsgade 2A, 1350 Copenhagen K, Denmark; katrine.rohder@psy.ku.dk; 6 The Family Clinic, Department of Obstetrics and Gynaecology, Amager and Hvidovre Hospital, Pavillon 4, Østre Hospitalsvej 5A, 2650 Hvidovre, Denmark; 7 Center for Clinical Research and Prevention, Bispebjerg and Frederiksberg University Hospital, The Capital Region of Denmark, Nordre Fasanvej 57, 2000 Frederiksberg, Denmarklotte.broberg.01@regionh.dk (L.B.)

**Keywords:** adverse childhood experiences, antenatal care, pregnancy, screening

## Abstract

Adverse childhood experiences have a potential lifelong impact on health. A traumatic upbringing may increase antenatal health risks in mothers-to-be and impact child development in their offspring. Yet, little is known about the identification of adverse childhood experiences in antenatal care. The objective of this study was to explore the feasibility and acceptability of the adverse childhood experiences questionnaire among midwives and factors affecting its implementation. Three Danish maternity wards participated in the study. The data consisted of observations of midwifery visits and informal conversations with midwives, as well as mini group interviews and dialogue meetings with midwives. The data were analysed using systematic text condensation. Analysis of the data revealed three main categories; “Relevance of the adverse childhood experiences questionnaire”, “Challenges related to use of the adverse childhood experiences questionnaire” and “Apprehensions, emotional strain, and professional support”. The findings showed that the adverse childhood experiences questionnaire was feasible to implement in Danish antenatal care. Midwives’ acceptability of the questionnaire was high. Training courses and dialogue meetings motivated the midwives to work with the questionnaire in practice. The main factors affecting the implementation process were time restrictions, worries of overstepping women’s boundaries, and a lack of a specific intervention for women affected by their traumatic upbringing circumstances.

## 1. Introduction

Over the last three decades, studies have consistently found that adverse childhood experiences (ACEs: abuse, neglect, and household dysfunction) during childhood and adolescence (up to the age of eighteen years) are associated with increased risk of physiological and mental illness in adulthood [1,2,3,4,5]. In European countries, it is estimated that approximately one out of four adults have had one adverse childhood experience, and one out of five adults have had two or more [1]. A large cross-sectional study has shown that among high-, high-middle-, and low-/lower-middle-income countries, a similar prevalence of ACEs in adults exists [6]. Compared to adults with no ACEs, adults with ACEs are more likely to smoke and have problematic drinking habits [7]. Correspondingly, adults with ACEs are also more likely to suffer from heart disease, respiratory disease, diabetes, and cancer, compared to adults with no ACEs [7].

The transition to motherhood poses a particular challenging period in life for women who have experienced a traumatic childhood [8,9]. Several studies have investigated ACEs among pregnant women to explore the potential associations between ACEs and risks during pregnancy as well as after birth [10,11]. One study has shown that two or more ACEs are associated with having an unwanted pregnancy [12]. ACEs are also associated with a higher risk of being in a relationship with domestic violence [13]. Several studies have documented a positive association between ACEs and mental illness, such as depression and anxiety during pregnancy or after birth [13,14,15,16]. Furthermore, Racine and colleagues have shown that ACEs are associated with antepartum health risks, defined by the authors as pre-pregnancy risk factors, past obstetrical risk factors, problems in the current pregnancy, and other risk factors [17]. At the same time, the potential negative health impact ACEs may pose in women during pregnancy may be buffered by resilience and social support [4,13,15,17].

After birth, studies point to ACEs as being associated with negative parenting behaviour [18] and parental stress [19]. These conditions can increase the risk of insecure attachment patterns in the offspring, and in more serious cases, lead to disorganised attachment [20]. Children with insecure attachment patterns, due to adverse childhood experiences, may replicate parental behaviour as adults when caring for children of their own, which may subsequently impact child development [9,21]. Thus, the early identification of ACEs and interventions are pivotal to decrease the risks of intergenerational transmission of parenting skills.

In their recommendations for antenatal care provision, the World Health Organization (WHO) highlights the importance of preventing or treating risks during pregnancy for the achievement of a positive motherhood, including maternal self-esteem and competence [22]. Despite recognition of the importance of early screening for ACEs and appropriate interventions [10,11], only a limited body of research has explored screening for ACEs within the antenatal care setting [11,23]. This is unfortunate, as screening during pregnancy may identify ACEs among women who may otherwise remain unrecognised for traumatic events during their upbringing. Information on women’s ACEs is important to increase maternity care providers’ awareness of the parenting challenges these women may endure after their child is born.

“The Invisibly vulnerable study” was developed to promote systematic screening for ACEs in Danish antenatal care and ensure adequate help for women, who due to their childhood circumstances need extra support during pregnancy and after birth. The study builds on previous qualitative research documenting adult daughters of alcoholic parents’ emotional challenges and concerns during pregnancy and the implications of these women’s experiences should have for antenatal care provision [24]. Exploring key stakeholders’ perspectives are important to understand how new work routines are received and operate in existing practices [25]. The present study is the first of two studies investigating midwives’ and women’s experiences with a 10-item ACE questionnaire [26] upon entry to midwifery-led antenatal care at three maternity wards in Eastern Denmark. The women’s experiences will be reported in a separate paper.

The objective of this study was to explore the feasibility and acceptability of the ACE questionnaire and factors affecting its implementation among midwives in Denmark.

### 1.1. Antenatal Care in Denmark

Danish antenatal care is publicly funded for all women who hold a residence permit [27]. According to the National Health Act, women have the right to at least five antenatal care visits during their pregnancy [28]. Antenatal care is structured with four referral levels [27]. Women assigned to level one are offered basic antenatal care usually comprising five to six midwifery visits, three visits with the general practitioner, and two ultrasound examinations [27]. Antenatal care level one targets women with expected uncomplicated pregnancies and is primarily provided by general practitioners and midwives. Levels two to four involve extended antenatal care services, such as additional antenatal care visits or prolonged visits. Level two targets women with antenatal, birth, or postnatal risks, i.e., women who are overweight, have had a previous complicated birth or breastfeeding problems—it is provided by maternity care providers. Antenatal care level three targets women with mental, medical, or social problems, while level four targets women with complicated problems relating to substance abuse, severe psychological or psychiatric disorders, or severe social disadvantages. Antenatal care in level three and four is provided by an interdisciplinary team usually including midwives, doctors, nurses, psychologists, psychiatrists, and social workers. Most women in Denmark are referred to antenatal care levels one and two [29]. For women with expected uncomplicated pregnancies, the midwife is the maternity care provider that the woman sees the most.

A screening for psychosocial vulnerability is placed during the first trimester of the pregnancy. At the first antenatal care visit, the general practitioner is responsible for confirming women’s pregnancy, as well as collecting and assessing women’s social, psychological, and physical history. Based on this information, the general practitioner refers the woman to the appropriate level of antenatal care [27]. Midwives follow up on women’s history the first time they see the woman.

### 1.2. The ACE Questionnaire in a Danish Antenatal Care Setting

Between June and October 2021, three hospitals in Eastern Denmark started to include questions on childhood experiences to their existing screening procedures in antenatal care as a quality improvement initiative. The new practice implied that all women assigned to antenatal care levels one and two were asked ten ACE questions by their midwife at the first or second midwifery visit, placed during the first or second pregnancy trimester. The number of births ranged from 1500 to 2400 per year at the three hospitals. Within the framework of this initiative, we designed a feasibility study, which is described in detail in the Materials and Methods Section 2.

The Adverse Childhood Experiences (ACE) Questionnaire is an internationally recognised questionnaire consisting of 10 questions about traumatic experiences in childhood (0–17 years) [30]. The instrument makes it possible to capture different categories of dysfunctional upbringing environments, and it has been found to be a strong predicative measure [2,10,30,31]. The WHO recommends that the tool should be deployed globally due to its effective predictive value and health-promoting potential [32]. A positive answer to a question adds one point to the respondent’s total score, i.e., between 0 and 10 points can be scored, with ≥4 considered serious [2,26,33]. Experience from other countries shows that it is possible to integrate the ACE questionnaire into prenatal care, and that successful implementation requires competence development and training of healthcare professionals as well as organisationally appropriate frameworks and resources [10,34].

The original ACE questionnaire was described in the Centers for Disease Control Kaiser Permanente adverse childhood experiences (ACE) study by Felitti and colleagues [30]. It focuses on a person’s experiences from birth to their 18th birthday and is composed of two clusters—one about different types of child maltreatment and another about household challenges. Instead of questions on different types of childhood adversities, the ACE questions centre around specific situations. For example, a question regarding psychological abuse asks about situations where an adult in the household “often or very often swear at you, insult you, put you down, or humiliate you or act in a way that made you afraid that you might be physically hurt” [26]. In this study, we used a validated version of the ACE questionnaire from the Centers for Disease Control and Prevention and Kaiser Permanente, where question items regarding emotional and physical neglect were added [26]. This questionnaire has been widely used in research [2,10].

A professional forward and backward translation of the questionnaire was carried out [35]. Further, we conducted a cultural translation with the intention of adapting the questions to Danish midwifery visits and to general conditions in Denmark [36]. A cultural translation is recommended by the WHO to enhance acceptability and cultural applicability of the questions, and appropriateness of wording and phrasing [37]. We used a WHO translation guideline regarding another health-related topic for inspiration [38]. We also had an expert group consisting of midwives with antenatal care experiences comment on the Danish version of the questionnaire. The cultural translation led to a slight change in the question order and to some minor modifications of the wording. Sub-items regarding household alcohol and drug abuse were merged into one question about “household substance abuse”, and information on incarceration, which had its own question, was included in a broader question about “loosing contact to a parent”. The final questionnaire included 10 questions on the following items: psychological abuse, domestic violence, physical abuse, sexual abuse, household substance abuse, lost contact to a parent/incarceration, parental separation, household mental illness/attempted suicide, emotional neglect, and physical neglect.

**Figure 1:**
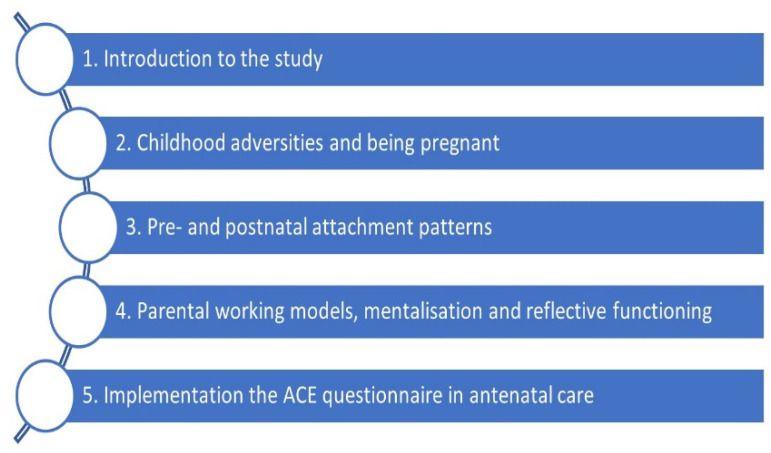
Training course themes.

Prior to the implementation of ACE in midwifery practice, fifty midwives providing antenatal care at the three hospitals’ maternity care wards received a one-day training course. A total of five training courses were held. To ensure access to training for all midwives, an online version of the course was offered to midwives who were unable to participate in the scheduled courses (number not recorded). Author V.d.L. facilitated both course types, which built on attachment theory, pedagogical theory, and existing research on childhood adversities. In the figure below, the course themes are presented (Figure 1).

Furthermore, midwives at the three maternity wards were offered participation in an online dialogue meeting where they were given the opportunity to share, discuss and receive feedback on their experiences and potential challenges related to implementing the ACE questionnaire. Author V.d.L. facilitated the dialogue meetings. In addition, an implementation manual describing how to introduce the questionnaire, ask the ACE questions, and follow up on women’s replies were provided for the midwives.

A local project midwife was assigned to each maternity ward to assist and monitor the implementation of the ACE questionnaire. The wards were compensated with 10 min to allow for extra time for women to complete the questionnaire verbally and for the midwives to introduce the questionnaire, record the women’s replies and follow up on the women’s ACE replies as well as their screening experiences. At one maternity ward, the ACE questionnaire was implemented into the existing time frame with no extra allocated time during the first four months of the study. Women were asked the ACE questions at the first or second midwifery visit during first or second pregnancy trimester. The midwives were instructed to offer women, who reported four ACEs or more, an extra midwifery visit. The women who scored between one and three ACEs were assessed for their need of an extra midwifery visit and offered one if relevant. The women who were identified as having severe mental or social problems due to their ACE history were offered a referral to antenatal care level three or four, where help from psychologists, psychiatrists and social workers was available with extra time resources allocated.

## 2. Materials and Methods

### 2.1. Design

The study was conducted as a feasibility study. According to Eldridge and colleagues, feasibility studies examine whether something can be carried out, whether it should proceed, and if so, how [39]? When investigating aspects related to feasibility and acceptability, a qualitative design is recommended [39,40]. Inspired by key principles from the evaluation research tradition, the study drew on multiple qualitative data sources to provide different perspectives of the phenomena [41]. The data sources consisted of observations of midwifery visits and informal conversations, as well as mini group interviews and dialogue meetings with midwives. Observations and informal conversations were performed from October 2021 to March 2022, mini group interviews from May to September 2022, and dialogue meetings January and February 2022. The data were collected from five antenatal care facilities.

### 2.2. Recruitment of Study Participants

Midwives for the feasibility study were recruited by the management or local project midwife. Inclusion criteria were being permanently employed as a midwife at one of the three maternity wards and undertaking midwifery visits in antenatal care. Midwives participating in the mini group interviews and dialogue meetings were paid a regular salary for their time.

### 2.3. Ethical Considerations

Midwives were informed verbally or in writing about the study prior to giving verbal consent to participate in observations or informal conversations. Midwives participating in the mini group interviews received written information before giving written consent to participate. Midwives participating in the dialogue meetings received verbal information about the study before verbally consenting to participate. All midwives contributing to the data collection were informed that their participation was voluntary, and they were guaranteed institutional as well as personal anonymity.

Since the ACE questionnaire might induce emotional reactions to previous trauma in some women, extra time was allocated for the midwives to brief and debrief the women. The midwives’ training course included communication training in facilitating difficult conversations with women affected by the questions. Additionally, the implementation manual contained suggestions on how to verbally prepare a woman for the questions, and how to address her experiences afterwards. The women who were emotionally affected by the screening process were offered an extra midwifery visit or referred to antenatal care level three.

In Denmark, certain types of research projects must be approved by a research ethics committee [42,43]. This applies to clinical trials and studies that involve human biological material. In the Committee Act, section 14, it is specified, that studies that do not involve human biological material should not be reported to the committee, and it is further specified that quality control and quality improvement initiatives should not be reported either [44]. The study, as well as a description of measures taken to ensure data protection, was reported to: The Research, Development, and Data Department, University College Copenhagen (ID number: 21-002). This department acts on behalf of the Danish Data Protection Agency [42].

### 2.4. Data Collection

#### 2.4.1. Observations and Informal Conversations

The structured observations (O) of midwifery visits were undertaken with the role of the observer as a participant, as described by Gold [45]. Such observer role calls for minimal involvement in the social setting of the midwifery visit. In all, 18 observations were performed. The observations lasted between 13 and 52 min. An observational guide was used to collect data [46]. It focused on the organisation of the antenatal visit, how midwives interacted with the women during the introduction to and completion of the ACE questionnaire, and how midwives followed up on the women’s ACE score. To further nuance findings, 9 h of informal conversations with midwives at the antenatal care facilities also contributed to the data. Continuous discussions of the observations and informal conversations within the author group allowed for further investigation of relevant themes throughout the study period. Observations and informal conversations were initially written in short form at the antenatal care facility. To ensure that they were described accurately and extensively, they were written in full as soon as possible after they had taken place. Observations and informal conversations were performed by author S.M.K.

#### 2.4.2. Mini Group Interviews and Dialogue Meetings with Midwives

Mini group interviews (MG) were chosen to ensure adequate time for the midwives to share and discuss their implementation experiences. Four mini group interviews were performed, three of these online. The interviews lasted between one hour and one hour and forty-seven minutes. A semi-structured interview guide was used to collect data [47]. The themes in the guide centred around the organisation of antenatal care at the local maternity ward, midwives’ experiences regarding the training course and dialogue meetings, and their work with the ACE questionnaire. Interviews were performed by authors H.J. and V.d.L. All interviews were audio-recorded and transcribed verbatim by a research assistant.

The dialogue meetings (DM) all lasted one and a half hours. The participating midwives decided themselves which themes were discussed during these meetings. Author V.d.L. facilitated all seven held dialogue meetings. A research assistant was responsible for documenting the dialogue meetings in writing.

### 2.5. Data Analysis

**Figure 2:**
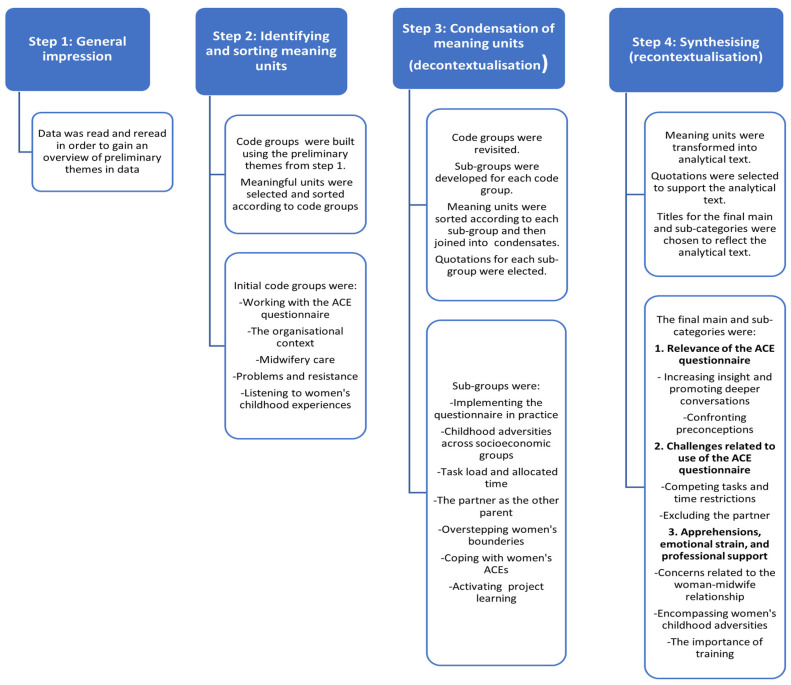
The four analysis steps.

Nvivo version 12 was used to store and manage the data [48]. The data were analysed using systematic text condensation, according to Malterud [49]. This method consists of four steps: (1) general impression; (2) identifying and sorting meaning units; (3) condensation of units and themes; and (4) synthesising. In Figure 2, the analysis process is described in more detail.

Observations, informal conversations, mini group interviews, and dialogue meetings were analysed with the same codes and then merged in step three of the text condensation process. Authors H.J. and V.d.L. undertook analysis step one. Author H.J. undertook analysis step two. The remaining analysis process was discussed among the authors to ensure that the categories and subcategories were built on the midwives’ narratives and were grounded in the different data sources included in the study.

## 3. Results

Fourteen midwives participated in the observations. Their professional experience varied from 1 to 21 years. Twelve midwives participated in the informal conversations (professional experience not recorded). Sixteen midwives participated in the mini group interviews. Their professional experience ranged from 1 to 39 years. Twenty-nine midwives participated in the dialogue meetings. Their professional experience ranged from less than 1 to 39 years.

Analysis of the data revealed three main categories with two or three subcategories, respectively. The main categories were “Relevance of the ACE questionnaire”, “Challenges related to use of the ACE questionnaire”, and “Apprehensions, emotional strain, and professional support”. The main categories and subcategories are elaborated below.

### 3.1. Relevance of the ACE Questionnaire

This category describes the midwives’ attitudes towards, and experiences of, working with the ACE questionnaire in antenatal care.

#### 3.1.1. Increasing Insight and Promoting Deeper Conversations

Overall, the midwives found the questionnaire to be a relevant screening tool. All described being previously unacquainted with the ACE questionnaire. The midwives explained how they, up until the implementation of the ACE questionnaire, primarily had focused on physical and mental conditions in adult life. Hence, vulnerabilities caused by trauma during women’s upbringing had not previously been routinely addressed during the midwifery visits. One midwife described how detailed questions about abuse contributed to a more comprehensive impression of the pregnant woman (M3, MG1). Another midwife argued that the ACE questionnaire could identify vulnerable women:

*“…It is a really good tool…these women were not referred to extended antenatal care services…they wouldn’t have been caught otherwise. It (the questionnaire) has accentuated, why it makes sense to ask them…” (M3, DM3)*

Some midwives explained that they saw the ACE questionnaire as a conversational tool, which contributed to legitimising conversations about issues of a more personal nature. This could promote confidentiality between the midwife and woman:

*“I see women, who open up, because they sense, this (the midwifery visit) is a room, where there is space to talk about difficult things.” (O9)*

Most midwives reported that the ACE questionnaire was well received by the women. According to the midwives, it was important to prepare the women for the ACE questions as well as taking the time to talk about women’s replies and their experiences of answering the questionnaire before ending the midwifery visit. One midwife described how her introduction to the questionnaire seemed to motivate the women to share their experiences:

*“…I don’t think I have had anyone who has refused it (the ACE questionnaire) …my experience is that women generally are very open, and they are happy to tell me what concerns them…it’s a good idea to explain why we think this (the questionnaire) is important…I have had some conversations that I have never had before.” (M4, MG3)*

According to the midwives, some ACEs could have a more profound impact on women than others. Thus, it was important for the midwives to investigate the impact of a particular ACE on a woman’s mental health. Women’s ACE replies were documented in their hospital records and thus also served to inform other settings in maternity care. Some midwives described sexual abuse cases as examples of the importance of awareness when providing targeted maternity care. This awareness was key to understanding women’s reactions and care needs:

*“…She had been sexually abused during several years of her childhood. It was her third child, and it hadn’t been previously identified in antenatal care…it was difficult for her to be touched and examined…she felt that midwives had reacted negatively towards her...I referred her to a midwifery team specialising in antenatal care for vulnerable women…” (M1, MG4)*

*“I asked her…what thoughts she had regarding breastfeeding, and if there was anything, we (the maternity care providers) should be aware of with regards to her body when starting to breastfeed…it was important for her that nobody used the hands on approach…It’s written in her birth plan.” (M1, MG1)*

The midwives described a few instances where a woman refused to answer the ACE questions. According to the midwives, the main reason for not wanting to answer the questionnaire was scepticism towards the maternity care system and concerns of being reported to the social authorities. A midwife described how vulnerability and insecurity had led a woman to refuse to answer the questionnaire:

*“…She had been referred to antenatal care level 2. I think, it should have been antenatal care level 3. It (the visit) was somewhat difficult. There was a lot of mistrust towards the system. She had thoughts of the social authorities becoming involved, being reported, she felt unsafe, didn’t want to open that box.” (M1, DM4)*

#### 3.1.2. Confronting Preconceptions

Several midwives described how they had been surprised that many women had a positive ACE score. Furthermore, a lack of information on women’s vulnerabilities in their hospital record was a frequent challenge:

*“It was an apparently completely normal second pregnancy, there was absolutely nothing to put a finger on…I tell her that I will proceed to ask her the ten (ACE) questions, she is fine with that…she answers no to the first questions, and then we get to if she has ever been sexually abused as a child and she answers yes…a friend of the family had raped her.” (M3, MG3)*

*“……Are you bringing any psychological issues with you, have you been exposed to stress?…I could hear there was something buried here….When I introduced the ACE questionnaire, she scored positive. Then her story came, it was long…Her doctor hadn’t recorded it...these women pop up in midwifery care…you can’t see what baggage the women bring by looking at them.” (M2, MG1)*

Several midwives explained how their estimation of a woman’s resources often were founded in impressions of the woman’s current personal and material resources, for example, her ability to communicate, her educational level, and her employment position. Nevertheless, experiences from working with the ACE questionnaire had shown the midwives that women with traumatic upbringing circumstances were represented across all socio-economic groups:

*“…what becomes really clear, is that childhood trauma is seen in all societal levels. I saw a woman who is a doctor…there were several things she scored on even though she is a doctor…You don’t connect it to that kind of position, it (the ACE questionnaire) covers a wide range of people.” (M1, MG3)*

*“…I have had some women…they scored high on everything, very surprising, because it wasn’t those, I would normally be able to pick out.” (M3, MG1)*

### 3.2. Challenges Related to the Use of the ACE Questionnaire

This category presents how organisational factors affected midwives’ implementation of the ACE questionnaire in practice.

#### 3.2.1. Competing Tasks and Time Restrictions

The main challenge was described to be the time available during the midwifery visits. This was due to a high task load combined with a restricted time schedule. Some midwives described how they would shorten some visits to free time for women with more complex antenatal care needs. However, as the antenatal care schedule was unpredictable, it was impossible to control:

*“I don’t find it difficult talking about it (the ACE questionnaire), but I am pushed on time. As a result, I am often late, and I use my lunchbreak on those women I didn’t get to.” (M2, DM 5)*

Compared to asking the ACE questions, following up on women’s ACE score was described as more uncontrollable because the midwives were unable to anticipate how long this conversation would take:

*“…I am nervous about opening the conversation about the ACE score because I don’t have the time.” (M2, DM6)*

Other midwives described how a lack of time to ask the ACE questions and follow up on women’s replies could occasionally result in the questionnaire not being used. Sometimes the women would have other care needs:

*“…the time pressure. Some issues can be more important…a previous birth, pelvic pain, a scan result…I must prioritise…Even though I have prepared the ACE questionnaire…sometimes it has not been possible to use it.” (M2, MG4)*

Midwives with only a few years of professional experience appeared more affected by restricted time resources than midwives who had worked in antenatal care longer:

*“…it can be a challenge, I am fairly new…if there is a lot the woman wants to talk about…we use all the time…it’s a shame when I get ten minutes extra to complete the (ACE) questionnaire.” (M4, MG1)*

#### 3.2.2. Excluding the Partner

Screening the woman for traumatic childhood experiences but not her partner was described as an issue by several midwives. Some midwives found that the lack of involvement of the partner resulted in unequal treatment. As the woman’s partner would become one of the primary caregivers for the expected child after birth, the midwives felt that the partner played an equally important role in establishing a healthy family:

*“…It’s frustrating…we tell the women this is important and then we disregard the partner…it seems wrong.” (O2)*

*“…it’s a shame the initiative is primarily directed toward the woman, the partners childhood is just as important when becoming a family.” (O11)*

As several women replied positively to the ACE questions, the midwives expected similar responses among their partners. Some midwives described how the women’s partners reacted when realising that the ACE questions were only directed towards the woman’s upbringing circumstances:

*“…The woman had scored six ACE points…the partner had said: my childhood was more violent, so it’s odd you don’t ask me.” (O10)*

*“The woman’s partner seemed uncomfortable when the woman had answered the ACE questions…He said: “I could say yes to eight of the questions. But there is no initiative for me.” (O12)*

### 3.3. Apprehensions, Emotional Strain, and Professional Support

This category illustrates midwives’ worries regarding to the ACE questionnaire, how they themselves were affected by women’s childhood trauma, and the role of education and training in supporting the midwives during the implementation process.

#### 3.3.1. Concerns Related to the Woman–Midwife Relationship

A few midwives expressed concerns that the ACE questionnaire might reinduce the trauma women had experienced growing up. They explained that they perceived pregnancy as an emotionally demanding period and thus they were worried that the ACE questions could induce psychological issues for women who were potentially vulnerable. One midwife described how a lack of professional autonomy regarding direct referral to a psychologist affected her concerns:

*“Maybe we will re-traumatise the women and what good is that when you can’t refer directly to a psychologist…” (M1, DM1)*

A few midwives described how a lack of training could potentially worsen women’s current situations:

*“…some women, if you address what has been hard, abuse, violence and so forth…they may need ten therapy sessions before they can verbalise it…If we move too fast, they may shut down…we are not psychotherapists or psychologists, we need to be very aware of that…” (M5, MG1)*

Another challenge described by the midwives was a lack of a specific intervention that targets women who are vulnerable due to adverse childhood experiences. They explained that some women needed an intervention which went beyond what the different antenatal care levels could offer:

*“…one of my concerns with the ACE questionnaire has been, what do we have to offer in cases where we think women’s problems are beyond our competencies…these women should be offered more help.” (M1, MG4)*

Many midwives found the ACE questions to be intimate. They also expressed anxiousness that subjecting women to the questions could damage their relationship with them because some women could experience the questions as overstepping their personal boundaries. One midwife explained how some women’s body language suggested that they found the ACE questions to be “too much” and “unnecessary” (O1). Other midwives felt different. They explained that the antenatal care visits already entailed several personal questions regarding women’s sexual and mental health and lifestyle. At the same time, establishing a relationship with the woman before they introduced the ACE questionnaire was important:

*“I prefer to establish a calm atmosphere before I move into their (the women’s) minds and private life.” (O6)*

#### 3.3.2. Encompassing Women’s Childhood Adversities

Sometimes the women’s histories entailed serious abuse and neglect. Several midwives described how they were used to “fixing” women’s problems. However, women’s childhood experiences could not be undone. Although the midwives had received training in how to work with childhood trauma in antenatal care, listening to the women’s history was described as very emotionally demanding by some midwives. One midwife described how she had felt overwhelmed by the number of women who scored high on the ACE questionnaire and how these midwifery visits felt burdensome (M4, DM5). Another midwife highlighted how she sometimes thought of her midwifery role as a caregiver who was expected to be able to deal with women’s history, no matter what she was told:

*“I have to be all encompassing…but sometimes I feel a little like a garbage bin, which can contain everything, we (the midwives) are also human beings.” (M3, MG1)*

One midwife reported how she felt she lacked training in coping with women’s experiences:

*“…It’s difficult questions you must ask. I can’t change what the women tell me they have experienced. But you need to be able to handle what they share…it’s not something we’ve learnt.” (M1, DM5)*

In addition, the midwives’ own life conditions were described to affect how they were able to cope with women’s stories. Some midwives explained how they as midwives experienced both periods with sufficient resources to cope with women’s issues and periods where they were challenged in their personal lives and thus less resourceful in dealing with them.

#### 3.3.3. The Importance of Support and Training

According to the midwives, having an implementation manual was very beneficial, especially at the beginning of the implementation period. Several midwives had used the manual as a support during their encounters with women. At the same time, some midwives found that the manual contributed to a more mechanistic communication with the women:

*“…reading them (the ACE questions) out loud made me more certain that the women gave the right answers…but I also felt it (the interaction) became impersonal…it made it more detached.” (M4, MG3)*

Receiving training in how to implement the ACE questionnaire was described as extremely important. Several midwives explained how the training course had increased their motivation to implement the ACE questionnaire as a new work routine. In addition, the training course had addressed some of the concerns the midwives had regarding women’s response to the ACE questionnaire:

*“…she (the teacher) said, they (the ACE questions) won’t make it worse for the women…Maybe they don’t see the connection…they may not think their childhood trauma can be of significance when they become a mother and that these issues may surface again…it (the ACE questionnaire) provided an opportunity to ask about the things we know are a little difficult to ask about.” (M2, MG3)*

In addition, discussions related to how to work with the ACE questionnaire in practice during the training course was described as very useful:

*“There were many usable phrases…the fact that we had to practice conversation techniques on how to introduce the ACE questionnaire…I thought that was really good. I am sure I have many colleagues who are more lost regarding the aim of the initiative, those who didn’t participate (in the training course).” (M4, MG3)*

Some midwives had not participated in the training course due to being on holiday, being off work ill, or not being employed at the time of the course. A few of them had used the online training course and the manual as teaching material instead. However, not having an opportunity to discuss the ACE questionnaire and ask questions was described to be a challenge, potentially leading to uncertainty in how to implement the questionnaire:

*“…I don’t get the same out of watching the online course. So I don’t really know how to approach it (the ACE questionnaire)…” (M2, DM3)*

*“I felt I was left on my own a lot…I found it hard to watch the online course. You can’t ask questions.” (M2, DM5)*

At one maternity ward, the time duration between the training course and the implementation of the ACE questionnaire in practice was approximately half a year. According to the midwives at this ward, the long time span impaired their memory of the training course.

Finally, the midwives described having mixed experiences from the dialogue meetings. Some midwives found that the dialogue meetings should have been scheduled at the beginning of the implementation period where their lack of experience with the ACE questionnaire was more pronounced. A need for more dialogue meetings as well as a need for personal supervision was mentioned by some of the midwives. Some midwives found the online form of the dialogue meetings to hinder discussions of their implementation experiences with other midwives. Other midwives reported that listening to how other midwives across the three maternity care wards worked with the ACE questionnaire contributed to new insights on how to work with the questionnaire in antenatal care:

*“I noticed how different the midwives worked with the ACE questionnaire. It was useful to see, how some midwives were really good at integrating the ACE questionnaire in the conversations they were already having with the women.” (M1, MG3)*

## 4. Discussion

Overall, midwives in this study found the ACE questionnaire to be a relevant screening tool, which indicates a high acceptability of the questionnaire in antenatal care. Among the perceived benefits was that the questionnaire created a safe space to talk to the women about sensitive subjects and helped the midwife to gain more comprehensive knowledge about who the woman was. This is in line with previous feasibility studies in maternal and childcare. Flanagan and colleagues reported that maternity care providers found that the ACE questionnaire contributed to building a rapport and trust with the women [34]. Hardcastle and Bellis found that health visitors reported that the ACE questionnaire improved their understanding of the women and their families as well as improving the quality and nature of their mutual relationship [50]. Likewise, Gillespie and colleagues found that paediatric primary health care providers described that the ACE questionnaire enhanced their empathy for their patients, bettered their understanding of and communication with their patients, and created a safe space to talk about their patients’ issues [51].

Midwives in our study also described that the women who scored positive on the ACE questionnaire varied significantly in their characteristics. Hence, they were sometimes surprised about which women reported traumatic upbringing circumstances. Similar findings are presented in a study showing that home visitors found it was not always those mothers that they expected to reveal the highest number of ACEs, who had a high ACE score [50]. The home visitors’ assumptions about the women were challenged and new information about the women was provided. Previous research has shown that the prevalence of ACEs in women is not directly associated with their income group [6]. In our study, the feasibility of the ACE questionnaire was explored exclusively in level one and two of Danish antenatal care. These levels provide antenatal care for women whose care needs are less complex. Even so, the midwives’ experiences suggest that some women were significantly impacted by their upbringing circumstances. Furthermore, these traumatic experiences had not previously been identified and recorded in the women’s hospital record. Thus, without screening for ACEs and assuring support, these women would likely have continued their antenatal care with these experiences tacit, potentially increasing their risks of perinatal depression [14,15,16] and parenting difficulties after birth [18,19].

Some challenges were described regarding the use of the ACE questionnaire. Following up on the ACE questionnaire in situations when women had a positive ACE score was described as time consuming by the midwives. In addition, midwives with less professional experience were generally more affected by time restrictions. Although compliance regarding implementation of the ACE questionnaire was generally high among the midwives, a few described situations where the questionnaire was not used. Related findings have been presented in paediatric care where care providers experienced time difficulties completing the ACE questionnaire [52]. Other studies have pointed to time resources to implement the ACE questionnaire as a smaller issue than anticipated [34,51]. These results highlight the importance of considering the local antenatal care context when investigating the questionnaire. Skivington and colleagues assert that in feasibility studies, the capacity to deliver an intervention may be impacted by factors related to the intervention’s implementation [53]. In our study, several factors impacted the questionnaires implementation at the three maternity wards. One ward did not allocate extra time to the screening process for the first four months and the implementation of the questionnaire started half a year after the midwives’ training course. Additionally, midwives’ time allocation as well as tasks varied between the first and second midwifery visit. It is likely that these factors affected the feasibility and acceptability of the questionnaire.

Another challenge described by the midwives was that the ACE screening was directed solely towards the mother and not the partner. In our study, only the ACE screening of mothers was possible with the funding resources available. In the recommendations for antenatal care, the Danish National Board of Health highlights the importance of promoting family-centred care and recommends, that antenatal care should support family formations and healthy attachment patterns between parents and their offspring [27]. Existing research from Nordic countries have shown that fathers’ care needs are often overlooked in antenatal care [54,55]. Thus, the inclusion of the partner in the ACE screening process is an important point, as the underreporting of ACEs among partners may be even higher than among the women due to the lack of psychosocial screening at the general practitioner.

A few midwives expressed concerns that the ACE questions might re-traumatize women with a positive ACE score. Traumatic childhood experiences are retrospective in adults, and thus the trauma related to living these experiences already exists. To the authors knowledge, there is no evidence in the existing literature that the ACE screening itself can trigger re-traumatisation [10]. On the contrary, a review on trauma-informed care suggests that the disclosure of past abuse in itself has the potential to be therapeutic [56]. At the same time, research within the trauma-informed care tradition has also shown that patients with a history of ACEs may experience re-traumatisation, if specific situations in health care resemble situations from their childhood [57]. These considerations are important for how maternity care providers interact with women who have had a traumatic upbringing. As highlighted by the midwives in our study, increasing awareness of potentially stressful situations, which may re-traumatise these women, is pivotal.

According to the WHO, addressing the emotional, psychological, and social needs of vulnerable women is an integrated part of routine antenatal care [22]. At the same time, the WHO emphasizes that antenatal care should be situated within a well-functioning health care system. Midwives in our study expressed concerns over the lack of an intervention for the women who due to their upbringing circumstances were vulnerable and needed extra support, even though the midwives could allocate extra visits to these women and/or refer them to specialised antenatal care services. Similar concerns have been voiced in another feasibility study of the ACE questionnaire in antenatal care where the clinicians noted the lack of mental health support resources available for women with ACE-related care needs [34]. Together, these findings accentuate the importance of ensuring care pathways (including targeted interventions) for women whose mental health is at risk due to their upbringing circumstances.

Many midwives considered the ACE questions intimate and expressed concerns about overstepping women’s personal boundaries. Several studies have addressed similar concerns in health care providers both in maternity care and other areas, and at the same time documented high acceptability of the ACE questionnaire among different patient groups [10,11]. Additionally, some midwives in our study found it emotionally demanding to listen to women’s narratives of their childhood trauma. These emotional difficulties may represent symptoms of work-related burnout, a condition where work is perceived as emotionally exhausting. Studies from Denmark, Sweden and Australia have reported considerable levels of work-related burnout among standard-care midwives [58,59,60]. Furthermore, it is probable that some of the midwives had endured ACEs during their own upbringing. As shown in a recent British study, midwives with ACEs themselves found the ACE screening process especially emotionally demanding [61]. In our study, some of the midwives expressed a need for supervision. Thus, offering group-based or individual supervision to midwives who conduct ACE screenings could be a means to overcome emotional burnout and emotional overload. In addition to supervision measures, some midwives may also need access to a psychologist to help them cope with the screening process.

Finally, the midwives highlighted the importance of training prior to implementation of the questionnaire. Similar tendencies have been reported in an earlier study showing that training can increase care providers’ knowledge about the consequences of ACEs including their relevance to prenatal health and increase skills in how to sensitively talk to patients about their ACEs [34]. Previous studies have also pointed to education and training as important tools to mitigate clinician discomfort during the ACE screening process [10,61]. Findings from our study showed that timing the training close to the actual implementation was important to the midwives, as was the staging of the training. The opportunity to discuss and share experiences during the training courses and dialogue meetings was highlighted as especially beneficial. These findings suggest that a constructivist learning perspective [62], where the midwives used their existing experience to gain knowledge and make meaning as a group, was useful in the implementation training, whereas individual online learning was perceived less effective.

### Main Limitations and Strengths

Main limitations include a variation between the three maternity wards regarding the timing of the ACE questionnaire (the first vs. second midwifery visit) as well as the time allocated to work with the questionnaire (10 min extra vs. no extra time). This is likely to have affected the midwives’ experiences and, thus, the findings. Additionally, three of the mini group interviews and all seven dialogue meetings were held online. This may have affected the interaction between the participating midwives and decreased the interviewers’/facilitators’ attention to non-verbal cues [48]. Finally, author V.d.L. facilitated the training courses, the dialogue meetings, and one mini group interview. This may have affected these midwives’ responses.

Strengths comprise the triangulation of data sources, data collectors, and data analysts, which contributes to the credibility of findings [41]. Also, the data were collected at three maternity wards with a total of five antenatal care facilities, which helped to nuance the findings. In addition, continuous collection and analysis of the data allowed for the assessment of sufficient information power in data [63].

Furthermore, the study findings may be transferable to similar organisations of antenatal care in other countries, i.e., Australia, Canada, France, the Republic of Ireland, the Netherlands, the United Kingdom, and New Zealand, which like Denmark, have shared antenatal care models [64].

Additionally, the implementation of the ACE questionnaire started during the COVID pandemic and the participating maternity wards had extraordinary problems regarding staffing shortages throughout the study period. We find it noteworthy that the tool gained an overall high acceptance among the midwives despite such challenges.

Finally, the study is one of the largest qualitative feasibility studies of the ACE questionnaire within the field of maternity care and childcare to this date. With its in-depth data, it makes a novel contribution to the limited evidence base regarding the questionnaire’s feasibility and acceptability in antenatal care.

## 5. Conclusions

Overall, the study findings show that it was feasible to implement the ACE questionnaire in Danish antenatal care. Training in the significance of ACEs during pregnancy and how to implement the questionnaire, as well as the opportunity to discuss and share implementation experiences, increased midwives’ motivation to integrate the questionnaire in the existing antenatal care practice. The midwives found that the ACE questionnaire contributed to a more comprehensive presentation of the woman and created room for dialogue about ACE-related vulnerabilities. These findings indicate high acceptability of the questionnaire among the midwives. Several factors were identified to affect the midwives’ work with the questionnaire. These included time restrictions, exclusion of the partner in the screening process, concerns about the intimacy of the questions, emotional strain coping with women’s childhood trauma, and a lack of a specific intervention for these women.

Future research is needed to explore how the organisation of antenatal care services can support care providers’ work with early identification of adverse childhood experiences among pregnant women. In addition, there is a need to further explore the development and efficacy of interventions that seek to prevent intergenerational transmission of impaired parenting skills caused by women’s childhood trauma.

## Data Availability

According to the General Data Protection Regulation, the qualitative data are confidential and cannot be provided.
